# Papillary Thyroid Carcinoma with Desmoid-Type Fibromatosis: Review of Published Cases

**DOI:** 10.3390/cancers13174482

**Published:** 2021-09-06

**Authors:** Abdallah Roukain, Stefano La Rosa, Massimo Bongiovanni, Marie Nicod Lalonde, Valérie Cristina, Michael Montemurro, Stephane Cochet, Alexandra Luquain, Peter A. Kopp, Gerasimos P. Sykiotis

**Affiliations:** 1 Service of Endocrinology, Diabetology and Metabolism, Lausanne University Hospital and University of Lausanne, 1011 Lausanne, Switzerland; abdallah.roukain@chuv.ch (A.R.); peter.kopp@chuv.ch (P.A.K.); 2 Institute of Pathology, Lausanne University Hospital and University of Lausanne, 1011 Lausanne, Switzerland; stefano.larosa@chuv.ch; 3 Synlab Pathology, 1003 Lausanne, Switzerland; massimo.bongiovanni@synlab.com; 4 Service of Nuclear Medicine and Molecular Imaging, Lausanne University Hospital and University of Lausanne, 1011 Lausanne, Switzerland; marie.nicod-lalonde@chuv.ch; 5 Service of Medical Oncology, Lausanne University Hospital and University of Lausanne, 1011 Lausanne, Switzerland; valerie.cristina@chuv.ch (V.C.); montemurro@web.de (M.M.); 6 Centre de Chimiothérapie Anti-Cancéreuse CCAC SA, Av. Alexandre Vinet 19b, 1004 Lausanne, Switzerland; stephane.cochet@ccac.ch; 7 Cypa Pathology, Unilabs, 1066 Lausanne, Switzerland; alexandra.luquain@unilabs.com

**Keywords:** papillary thyroid cancer, desmoid-type fibromatosis, nodular fasciitis-like stroma, BRAF, CTNNB1

## Abstract

**Simple Summary:**

Papillary thyroid cancer (PTC)-desmoid type fibromatosis (DTF) is one of the rarest variants of PTC. The diagnosis is histological, and detecting a mutation of CTNNB1 in the mesenchymal component is highly suggestive of PTC-DTF. The treatment is essentially surgical. We conducted a review of all cases of PTC-DTF found in the English literature and our aim is to describe patient’s characteristics, histology, immunohistochemistry and somatic mutations of every case.

**Abstract:**

Desmoid-type fibromatosis (DTF) is a very rare variant of papillary thyroid carcinoma (PTC). It is essentially a dual tumor with a component of classical PTC with malignant epithelial proliferation (*BRAF*-mutated) and another component of mesenchymal proliferation (*CTNNB1*-mutated). We conducted a literature review on PTC-DTF. In total, 31 articles were identified, that together reported on 54 patients. The mean age was 47 years, with a 2.2:1 female predominance. No ultrasound features were found to be helpful in differentiating PTC-DTF from other PTC variants. Of the 43 cases that reported histological details, 60% had locally infiltrative disease (T3b or T4). Around 48% had cervical lymph node metastases, but none had distant metastases. While PTC-DTF may be locally more aggressive than classic PTC, its overall behavior is similar and can include extrathyroidal extension and lymph node metastases, which may contain a stromal component and show extranodal invasion. The mainstay of treatment for PTC-DTF is surgery, and the DTF component is not expected to be sensitive to radioactive iodine. External radiotherapy, non-steroidal anti-inflammatory drugs, tyrosine kinase inhibitors and chemotherapy have also been used in selected cases. Due to the rarity of these tumors and the lack of specific treatment guidelines, management should be discussed in a multidisciplinary team.

## 1. Introduction

Papillary thyroid carcinoma (PTC), follicular thyroid carcinoma, Hürthle cell carcinoma, and their histological variants, represent differentiated types of thyroid carcinoma (DTC), and account for the vast majority (>90%) of thyroid cancers [1]. In 2009, DTC had a yearly incidence of 14.3 per 100,000 in the United States of America (USA) [1], and this incidence has been increasing, mainly due to an increase detection of PTC [1]. PTC is the most frequent type of thyroid cancer, and comprises several variants (classical, follicular, tall-cell, columnar, hobnail, cribriform-morular, solid-trabecular, diffuse-sclerosing, among others). Several of the non-classical variants are considered to be clinically more aggressive. One of the rarest variants is PTC with desmoid-type fibromatosis/fasciitis stroma (DTF), representing 0.03–0.5% of PTCs and described only as case reports or small case series. The first cases were reported in 1989 by Ostrowski et al. [2], using the term “histological myxomatous changes”, and in 1991, as PTC with “nodular fasciitis-like stroma” [3]. Since then, the cases reported in the literature have been designated with various terms including “fibromatosis-like stroma”, “nodular fasciitis-like stroma” and “myofibroblastic stroma”. Histologically, this PTC variant is essentially a dual or biphasic tumor. One component consists of a malignant epithelial proliferation with typical features of classical PTC, whereas the second component is characterized by a mesenchymal proliferation, including fibroblasts and myofibroblasts, that resembles nodular fasciitis or fibromatosis [4]. The vast majority of cases described in the literature comprise >80% mesenchymal cells and <20% malignant thyroid follicular cells [4]. In 2017, based on the demonstration that each of the tumor’s components harbors a distinct somatic mutation (i.e., *BRAF* mutation in the epithelial component and *CTNNB1* mutation in the mesenchymal component), we proposed the term “papillary thyroid carcinoma with desmoid-type fibromatosis” (PTC-DTF) [4]. In the present review, we list and discuss all 54 patients previously reported in the English literature (Table 1).

## 2. Literature Review and Discussion

A review of the literature was conducted in December 2020 in the Pubmed database, limited to publications in the English language, and using the following search strategy: thyroid carcinoma AND (desmoid-type fibromatosis OR nodular fasciitis-like stroma OR fibromatosis-like stroma). The literature search yielded 60 publications. We retained 26 relevant publications based on screening of the abstracts; 5 more were identified after screening the references from the literature search results. Thus, a total of 31 publications were included, comprising a total of 54 reported cases of PTC-DTF (Table 1). The first case was described by Ostrowski et al. in 1989 and was referred to as “myxomatous change in papillary carcinoma of thyroid” [2]. In 1991, Chan et al. described three cases of PTC with “nodular fasciitis-like stroma” [3]. Chan et al. [3] and Mizukami et al. [5] reported the incidence of this PTC variant to be 0.5% and 0.17%, respectively, of all PTC cases diagnosed in their respective centers. In 2017, we reported the incidence of this variant to be 0.03% of all PTC cases at the Lausanne University Hospital [4]. These numbers define PTC-DTF as one of the rarest variants of PTC.

Although fibromatosis and nodular fasciitis share certain similar features such as cytologically bland spindle cells, they are two different entities, each with a distinct clinical course: nodular fasciitis is considered self-limiting and has the possibility to regress spontaneously, whereas fibromatosis tends to behave more aggressively [4]. Morphology alone is sometimes unable to distinguish the two entities; in such cases, specific genetic alterations can facilitate the differential diagnosis because nodular fasciitis can harbor *USP6* rearrangements, while fibromatosis *CTNNB1* mutations [34].

DTF is (myo)fibroblastic, locally aggressive and frequently recurring soft tissue neoplasm that develops in musculo-aponeurotic structures, favoring the extremities, the joints, the abdomen, the head and the neck [35,36]. Some cases occur in patients with familial adenomatous polyposis (FAP) [35], who also have a higher risk of developing the cribriform-morular variant of PTC. The overall incidence of DTF is about 2.4–4.3 per million per year [35], with 1000 new cases diagnosed per year in the USA [36]. Tumor cells are positive for smooth muscle actin (SMA) and show nuclear expression of β-catenin, reflecting *CTNNB1* mutations [35] as demonstrated in 89% and 92% of DTFs in two series of 204 and 117 patients, respectively [37,38].

There is no universally accepted terminology for these tumors. The DTF component was predominant in the most of reported cases (up to 95% in some cases), while others contained around 50% of PTC. Rebecchini et al. [4] proposed that cases of PTC with a prominent mesenchymal component should be named “PTC with DTF”. Conversely, for tumors with a predominant DTF component, “DTF with PTC” might be more appropriate. However, neither of these terms highlights the neoplastic nature of the DTF component.

As seen in Table 1, age at diagnosis of PTC-DTF varied from 19 to 82 years, with a mean age of 44.8 years and a median age of 43 years. There was a predominance of women (female-to-male ratio of 2.2:1), a characteristic of thyroid carcinoma in general. Of the 45 cases with available data, 61% had locally infiltrative disease (T3b or T4); this involved either the PTC component, the DTF component, or both. Most cases presented an infiltration of strap muscles (T3b according to the 8th Edition of the American Joint Committee on Cancer 2017 Cancer Staging Manual [39]), but some cases also showed infiltration of subcutaneous soft tissues (T4a [39]), and Michal et al. described one patient with parathyroid infiltration by the thyroid tumor [6]. Based on these observations, it appears that PTC-DTF may have a higher risk of locally infiltrative disease compared to classical PTC.

About 48% of patients had lymph node metastases, which is towards the higher range of what has been reported for other DTC variants (20–50%), and is closer to aggressive variants of PTC (such as the tall-cell variant). [1]. For comparison, Giani et al. [40] found 26% of lymph node metastasis in patients with classical PTC and 9% in patients with follicular variant of PTC, whereas Longheu et al. [41] reported 45% lymph node metastases in patients with tall-cell variant of PTC. The largest series of PTC-DTF by Takada et al. described 14 cases [28]. Metastatic lymph nodes were detected in 12 of the 13 cases that underwent lymph node dissection; only in the central neck (N1a) in five cases and in both the central and lateral neck (N1b) in seven cases [28]. In four cases, lymph node metastases harbored a DTF component (mesenchymal tumor cells) with or without a PTC component (epithelial tumor cells), while the other cases contained only a classical PTC component [28].

Among studies reporting tumors that were locally invasive (T3b/T4) or metastatic to lymph nodes (N1), several specified what component was responsible for the local invasion or lymph node metastasis, respectively. Among T3b/T4 tumors, in most cases, the DTF component was responsible for gross extrathyroidal extension; Wong et al. [31] reported extrathyroidal invasion by both components. Among N1 tumors, most cases reported presence of the PTC component in lymph nodes; Takada et al. [28] reported presence of the DTF component in 4 of 12 cases, with or without the PTC component (Table 1).

Most of the cases reported in the literature had a relatively short follow-up of around 1 to 2 years, and recurrences have been rarely described. The following cases illustrate that recurrences do occur and that long-term follow-up is warranted in patients with PTC-DTF. In a patient with locally invasive PTC-DTF (initial stage T4a N0 M0) reported by Zhou et al. [30], initial treatment consisted of thyroidectomy with ipsilateral central neck dissection, followed by radioactive iodine (RAI) therapy. Six months later, locoregional recurrence was detected and followed with ultrasound imaging for 2 years, at which time the residual tumor size was 4.5 cm. A second operation was performed and the tumor was found to be invading the strap muscles, trachea and esophagus (T4a). After an extended resection, the patient refused external beam radiotherapy and remained free of disease on last follow-up (2 years later) [30]. Khalil et al. also reported a recurrence of a PTC-DTF, 1 year after the initial surgery [22]. Before the first operation, the tumor measured 3.5 cm in largest dimension (T2), and the operation was followed by RAI therapy. Recurrence was detected during a pregnancy. After the patient delivered, a diagnostic whole-body RAI scan did not show uptake by the neck mass; she was then treated with surgery followed by antiestrogen therapy (tamoxifen 20 mg twice daily) with partial response (remnant of 1 cm) during a 2-year follow-up [22]. The authors justified the use of tamoxifen by referring to the “temporal relationship of the tumor with pregnancy”, and they also documented positive immunostaining for estrogen receptors in the tumor cells (original tumor and recurrence). Of note, tamoxifen is contraindicated during pregnancy and lactation.

Tajiri et al. [42] described the ultrasound characteristics of 13 patients with PTC-DTF. Based on sonographic criteria, 2 of the nodules were categorized as intermediately suspicious and 11 as highly suspicious for malignancy prior to surgery. The mean diameter of the index lesions was 37 mm (range: 16 to 79 mm), 11 nodules were “taller than wide”, and 46% had ill-defined margins. All nodules were irregular in shape and heterogeneous in aspect, with 39% being markedly hypoechoic, 23% hypoechoic and 38% isoechoic; in addition, 23% harbored microcalcifications. The authors concluded that PTC-DTF cannot be differentiated from classical PTC based on ultrasonography, which is the case for most PTC variants, with some exceptions such as the diffuse-sclerosing variant [43]. Our previous study described the ultrasound characteristics of 2 patients with PTC-DTF [4]. One patient showed a partially cystic nodule of 38 mm with microcalcifications. The other patient showed an ill-defined 28 mm nodule without microcalcifications.

The diagnosis of PTC-DTF is challenging on fine-needle aspiration (FNA) samples. Takada et al. [28] reported 12/13 cases as suspicious for malignancy or malignant. The cytology of one case was reported as benign, and schwannoma or fibroma was suggested [28]. Our previous study [4] reported the cytopathology results for two patients. In both cases, mesenchymal cells were observed in the cytology material, but this component was not considered relevant for the initial diagnosis. Mesenchymal cells can be seen in thyroiditis (Hashimoto’s and Riedel’s thyroiditis), as well as in tumors such as anaplastic thyroid carcinoma, medullary thyroid carcinoma, or sarcoma [11,33].

It is important to avoid misinterpreting the fibrotic component as benign reactive fibrosis. Moreover, the spindle cells may be mistaken for transformation into anaplastic thyroid carcinoma, and *CTNNB1* mutation can also be present in anaplastic thyroid carcinoma [44]. However, unlike PTC-DTF, anaplastic thyroid carcinoma can show coagulative necrosis of blood vessels, scattered atypia and mitosis at the periphery of the fibrosis, and blood vessels obliterated by spindle cells.

In addition to the PTC-DTF cases listed in Table 1, Cho et al. [45] recently described the first case of a medullary thyroid carcinoma with DTF in a 36-year-old woman. The DTF component had infiltrated the perithyroidal soft tissue, and five lymph nodes harbored metastases with areas of DTF. Similar to PTC-DTF cases, Cho et al. observed a nuclear expression of β-catenin in the DTF component [45].

Immunohistochemical results of PTC-DTF are summarized in Table 1. The PTC component is characterized by positivity for thyroglobulin, thyroid-restricted transcription factors (PAX8, TTF1), cytokeratin and membranous β-catenin in the PTC components (Table 1). In contrast, the DTF component tends to express markers such as nuclear and cytoplasmic β-catenin, vimentin and SMA, but expression of proteins that define thyroid follicular cells (thyroglobulin, PAX8, TTF1) is absent.

Mutational analyses have provided definitive evidence for the biphasic nature of PTC-DTF (Table 2). The PTC component can harbor a somatic activating mutation in *BRAF*, most commonly c.1799T > A (p.V600E) [1,4], more rarely c.17991801delTGA (p.V600K601delinsE) [31]. Overall, a *BRAF* mutation can be found in about 45% of cases of PTC [46]. In contrast, activating mutations in *CTNNB1* have been identified in the DTF component and currently include c.133T > C (p.S45P) [4,33], c.121A > G (p.T41A) [29,33], and c.134C > T (p.S45F) [31]. Interestingly, somatic mutations in *CTNNB1* have also been reported in some cases of the cribriform-morular variant of PTC [4].

Due to the rarity of PTC-DTF, no specific management guidelines exist in the literature. Management principles can be extrapolated from those applicable to other forms of DTC [1], as well as extra-thyroidal desmoid tumors [48]. Asymptomatic patients with extra-thyroidal desmoid tumors do not always require treatment, as retrospective series show a progression-free survival rate of 50% at 5 years [48]. If treatment is indicated, the first step of management is surgery, ideally with resection-free margins. Adjuvant radiotherapy can be considered for symptomatic patients with progressive or recurrent tumors [49]. Systematic treatment options include anti-hormonal therapy (tamoxifen), non-steroidal anti-inflammatory drugs (e.g., sulindac, etodolac), tyrosine kinase inhibitors (e.g., imitanib, sorafenib, sunitinib, pazopanib), low-dose chemotherapy (e.g., methotrexate and/or vinblastine/vinorelbine) or full-dose chemotherapy (e.g., doxorubicin) [48,49]. Anti-hormonal therapy has been described in case reports, and no head-to-head study was conducted with tamoxifen versus another treatment.

The rate of distant metastasis in PTC variants ranges from 1% for the encapsulated follicular variant of PTC to 15% for the solid variant and the diffuse-sclerosing variant [1], and overall distant metastasis rates for DTC are reported to be 5–23% [50]. Interestingly, none of the patients with PTC-DTF reported in the literature developed distant metastases (Table 1). One consideration is that, because most of the tumor volume is comprised by the DTF component, this may contribute to a lower metastatic potential compared to other PTC variants, since desmoid tumors are not known to have distant metastases. Another consideration is that the follow-up in many of the articles is rather short (a few years).

## 3. Conclusions

PTC-DTF is a very rare variant of PTC, with only 54 cases reported in the English literature. This variant may show a more aggressive behavior compared to classical PTC in terms of local infiltration and with a relatively high risk of lymph node metastases, but without any known cases of distant metastases to date. PTC-DTF is clinically and radiologically difficult to differentiate from other PTC variants. On FNA samples, a mesenchymal component is sometimes described, but it might not be considered relevant for the diagnosis, or its presence might lead to a misdiagnosis of schwannoma or sarcoma. Immunohistochemistry with positivity for nuclear β-catenin and the presence of somatic mutation of *CTNNB1* in the DTF component can be helpful to establish the diagnosis of PTC-DTF.

Clinicians should be aware of this variant, which needs to be included in the differential diagnosis of a rapidly growing thyroid nodule. For PTC-DTF, the first-line management is surgery aiming for a total resection; this is important because, in contrast to the classical PTC component that may respond to RAI, the DTF component should be considered RAI-refractory due to its mesenchymal nature. Patients with advanced or progressive PTC-DTF tumors should be managed by multidisciplinary teams with experience in the management of thyroid carcinomas and sarcomas including endocrinologists, high-volume surgeons, oncologists, radiologists and nuclear medicine specialists.

## Figures and Tables

**Table 1:** Reported cases of PTC-DTF.

Authors	Year	No of Cases	Age	Sex	Invasive (T3b/T4)	N1	M1	IHC-PTC	IHC-DTF
Ostrowski et al. [2]	1989	1	54	♂	no	nr	no	Tg +	Ker +
Chan et al. [3]	1991	3	20 35 20	♀ ♀ ♀	no no no	no no no	no no no	Tg, CKer, Vim, S-100 + *	Vim, MSA, Des + *
Mizukami et al. [5]	1992	1	67	♀	no	no	no	Tg +	Vim +; Lys, Des, S-100 −
Michal et al. [6]	1992	2	49 33	♀ ♀	yes ^a^ yes ^a^	yes ^b^ no	no no	Tg, CKer + *	Act, Des + *
Mizukami et al. [7]	1995	1	43	♀	yes	yes ^b^	no	Tg, CKer +; Cal −	Des, Vim +; S-100, MSA −
Terayama et al. [8]	1997	1	57	♀	nr	no	no	nr	Des, Vim, MSA +; CKer, Tg, S-100 −
Acosta et al. [9]	1998	1	41	♀	yes	no	no	Vim +; Act −	Vim, MSA +; Tg, CKer −
Sarma et al. [10]	1998	1	50	♀	nr	nr	nr	Tg +	nr
Yang et al. [11]	1999	1	82	♂	nr	no	no	nr	Vim, MSA +; Tg, CKer −
Toti et al. [12]	1999	2	73 24	♂ ♀	yes yes	yes ^b^ yes	no no	Tg, CKer + *	Vim, MSA, Des, TGF-β + *
Us-Krasovec et al. [13]	1999	1	42	♀	nr	no	no	nr	Vim, Act, MSA, Des +; S-100 −
Naganuma et al. [14]	2002	2	52 40	♂ ♂	yes yes	no yes ^b^	no no	nr	Vim, MSA +; Tg, S-100, Des − *
Inaba et al. [15]	2002	2	65 26	♀ ♀	no no	no no	no no	Tg −, CKer, Vim + *; Tg, CKer, Vim + *	Vim, MSA +; S-100, Des − * Vim +; Des, SMA, S-100 − *
Andres et al. [16]	2005	1	35	♀	no	no	no	nr	nr
Lee et al. [17]	2006	1	40	♀	nr	no	no	Tg, CKer, Vim +	Vim, MSA +; CKer, Tg, Des −
Basu et al. [18]	2006	1	35	♀	no	no	no	nr	nr
Leal et al. [19]	2008	1	34	♀	no	no	no	nr	MSA +
Lee et al. [20]	2008	1	45	♀	nr	yes	no	nr	nr
Lee et al. [21]	2010	1	35	♀	yes	yes	no	nr	nr
Khalil et al. [22]	2010	1	29	♀	yes ^a^	nr	no	Tg, CKer, TTF-1 +	Vim, MSA, Des +; CKer, S-100 −
Nandeesh et al. [23]	2011	1	46	♀	yes	no	no	nr	nr
Na et al. [24]	2013	1	49	♀	nr	yes ^b^	no	β-catenin, TGF-β +	β-catenin, TGF-β, Des, MSA +; Tg, CKer −
Wu et al. [25]	2013	1	42	♀	nr	no	no	nr	Vim, MSA +; Tg, S-100 −
Ginter et al. [26]	2015	1	44	♀	nr	yes ^b^	no	TTF-1+	MSA, Des, β-catenin −
Mardi et al. [27]	2017	1	38	♀	nr	no	no	nr	Vim, MSA +; CKer, Tg −
Rebecchini et al. [4]	2017	2	34 48	♂ ♂	yes ^a^ no	no no	no no	Tg, Vim, m β-catenin + Tg, m β-catenin +; Vim −	*n*, c β-catenin, Vim, MSA + *n*, c β-catenin, Vim, MSA +
Takada et al. [28,29]	2017, 2018	14	49.3 **	9♀, 5♂	9/14	12/13 ^a,b^; 1/13 nr	no	m β-catenin + *	*n*,c β-catenin + *
Zhou et al. [30]	2018	1	48	♀	yes	no	no	c β-catenin, Tg +	*n* β-catenin +, Tg −
Wong et al. [31]	2019	1	58	♂	yes ^a,b^	yes	no	*n*,c β-catenin +; SOX11 −	*n*,c β-catenin, SOX 11 +
Roth et al. [32]	2019	1	20	♂	yes ^a^	no	no	m β-catenin, TTF-1 +	*n* β-catenin, MSA +
Suster et al. [33]	2020	4 #	53 65 30 58	♂ ♂ ♀ ♂	yes yes yes no	nr nr nr nr	nr nr nr nr	3/4: m β-catenin, SMA − 1/4: nr	2/4: *n* β-catenin + 1/4: *n* β-catenin − 3/4: SMA + 1/4: nr
Total		54	43.9 **	2.1:1 ***	27/44	22/46	0/48		

Act: actin; c: cytoplasmic; Cal: calcitonin; CKer: cytokeratin; Des: desmin; DTF: desmoid-type fibromatosis; IHC: immunohistochemistry; Ker: keratin; Lys: lysozyme; m: membranous; MSA: muscle-specific actin; *n*: nuclear; nr: not reported; PTC: papillary thyroid carcinoma; S-100: S-100 protein; Tg: thyroglobulin; TGF-β: transforming growth factor beta; TTF-1: thyroid transcription factor 1; Vim: vimentin; −: negative; +: positive; ^a^: DTF component; ^b^: PTC component; *: there was no information on how many cases were positive or negative in IHC; **: mean; ***: female-to-male ratio; #: this study analyzed a total of 7 patients, two of whom were previously reported and genotyped by Rebecchini et al. [4] and one was previously reported by Roth et al. [32].

**Table 2:** Somatic mutations reported in PTC-DTF.

Authors	Somatic Mutations
Ginter et al. [26]	BRAF V600E *
Rebecchini et al. [4]	PTC: BRAF V600E (2/2) DTF: CTNNB1 S45P (2/2)
Takada et al. [28,29]	PTC (7 cases analyzed): BRAF V600E (7/7) DTF (8 cases analyzed): CTNNB1 T41A (1/8)
Zhou et al. [30]	DTF: CTNNB1 S45F
Wong et al. [31]	BRAF V600_K601delinsE * CTNNB1 S45P *
Roth et al. [32]	DTF: CTNBB1 T41A, reported in Suster et al. [33]
Suster et al. [33] ^¶^	PTC: BRAF V600E (in 2 cases of 4), NRAS A59T (1/4) DTF: CTNNB1 S45F (in 1 case of 4), CTNBB1 T41A (2/4) ^#^

*: These studies did not specify the tumor component (epithelial or stromal) in which the respective mutations were present. ^¶^: the results of the previously reported and/or analyzed patients are indicated in the Table 1 and Table 2 in the respective original studies. Among the other patients, one (the 65-year-old male) had a tumor that harbored a BRAF V600E mutation and was negative for CTNBB1 mutations. ^#^: In the text of this paper, it is mentioned that “three cases showed a CTNNB1 c.121A > G (p.T41A) mutation”. However, the last figure of that paper actually indicates two cases with a CTNNB1 c.121A > G (p.T41A) mutation and a third case with a CTNNB1 c.124A > G (p.T41A) mutation. As nucleotides 121 and 124 belong to different codons, they cannot both affect the same amino acid residue (T41). Unfortunately, because threonines are present at positions 40, 41 and 42 of the CTNNB1 protein, it cannot be concluded from the information available in the paper where the error lies, and which of the two contradictory affirmations is accurate [47].
